# ERRATUM

**DOI:** 10.1590/1984-0462/;2018;36;3;011016erratum

**Published:** 2018

In the manuscript “Physical education classes and health outcomes in brazilian students”, DOI: 10.1590/1984-0462/;2018;36;2;00011, published in the Rev. paul. pediatr. 2018;36:192-8.

Where it reads:

^c^Brunel University, Uxbridge, Reino Unido.

It should read:

^c^Brunel University London, Uxbridge, Reino Unido.

In the manuscript “Ultrasonographic markers of cardiovascular disease risk in obese children”, DOI: 10.1590/1984-0462/;2018;36;2;00016, published in the Rev. paul. pediatr. 2018;36:171-5.

Where it reads:

Carlos Alberto Nogueira-de-Almeida^a^

^a^Faculdade de Medicina de Ribeirão Preto, Universidade de São Paulo, Ribeirão Preto, SP, Brazil.

It should read:

Carlos Alberto Nogueira de Almeida^b^

^b^Departamento de Medicina, Universidade Federal de São Carlos, São Carlos, SP, Brazil.

